# On the Use of Chloroform in Infantile Convulsions

**Published:** 1870-05

**Authors:** David Mackintosh

**Affiliations:** Late President of the Hamilton (Ontario) Medical and Surgical Society, and one of the Physicians to the Hamilton City Hospital.


### SELECTED ARTICLES.

### ARTICLE IV.

### *On the use of Chloroform in Infantile Convulsions.*

By David Mackintosh, M. D., L.R.C.S. Edin.,

Late President of the Hamilton (Ontario) Medical and Surgical Society, and one of the Physicians to the Hamilton City Hospital.

(A paper read before the Canadian Medical Association at its meeting held in Toronto on the 8th September, 1869.)

To be awoke during the night to find a child, who had gone to bed apparently in the best of health, racked with convulsions and distorted by spasms, or during the day to observe a child who has been running about, in all the joy and buoyancy of youth, and in the very flush of health, suddenly fall, become unconscious, and in an instant be disfigured with the twitchings or the powerful contractions peculiar to convulsions, is one of the most painful and startling positions in which a parent can be placed, and in which, almost more than any other, he looks to the medical man for help. To be summoned to such a case, and to be able to do little or nothing likely to give certain relief, where so much is expected of him, is one of the most trying positions in which a Physician can be placed.

Every member of our Profession must be aware that in such a case he has to stand idly by, till, the source of irritation having been removed by the efforts of nature, the patient rallies, or till the exhausted frame sinks under the strain on the nervous system, and death comes to the relief of the little sufferer, or make use of remedies in which he has little faith, and which are mostly employed from a conviction that *something* must be done to satisfy the relatives of the patient, however small the prospect of doing good.

In such cases the difficulty is not so much the want of remedies calculated to give relief as the impossibility of administering them in the ordinary manner, since all the avenues usually employed for the introduction of these remedies into the system are closed, the jaw is locked, and the power of deglutition gone, and the anus is relaxed, and thus unable to retain enemata.

In such cases the practice generally has been to plunge the patient into a warm bath, while the head is kept cool with ice or vinegar and water or the cold water douche, to apply sinapism or some other rapid and powerful counter-irritant to the lower part of the body, administer, or attempt to administer, enemata, either with the view of working out the irritating matter from the primæ viæ, or to act as anti-spasmodics, and in some cases, (*and that more to have the appearance of not being idle*) to apply leches.

If, however, we look at the rationale of the cause of such convulsions in most cases (and let it be distinctly understood that this paper refers to convulsions arising primarily either from the irritation of teething or from the presence of indigestible substances in the stomach or bowels and not to convulsions caused by disease of the nervous centres) if, I say, we look at the explanation of such cases we will see that most of these modes of procedure are either injurious or altogether useless.

Take, for instance, a case of convulsions arising from the irritation of teething, we find that the presence of the advancing teeth stretch the resisting gums and irritate the delicate nervous fibres, and thus in all cases causes a great amount of irritability, and, in very susceptible constitutions, this irritation being conveyed to the centre of the excito-motary system, causes, by reflex action, irritation of the motary nerves, the visible evidence of which we have in alternate contraction and relaxation, of the parts supplied by these nerves, termed spasms or convulsions according to their extent or duration.

On the other hand take a case of convulsions arising from the presence of indigestible matter in the primæ viæ, we have direct irritation on the o organic nerves which like a delicate net work cover the intestines—this irritation passing along the large ganglia, to the excito-motary system, and producing, by reflex action, the convulsions and spasms already referred to.

In both instances, of course, the removal of the source of irritation must be a primary object, and where this can be done quickly and thoroughly at the very commencement of the spasms, and that more especially in some children (who not having those finely strung nerves which seem to be peculiar to certain families, and which render them subject to convulsions, from very slight sources of irritation) the disease is very often checked at once and the patient cured. Thus, for instance, in convulsions from teething, timely scarification of the gums will often stop the spasms, while in cases of convulsions from irritation of the bowels, if the first attack be slight, and we have time for the administration and action of drugs calculated to clear out the primæ viæ, before a recurrence of the muscular contractions, we may often relieve the patient. But how frequently does it happen in these cases, and more especially in those that prove fatal, that the jaw remains locked and the power of deglutition gone, preventing on the one hand the use of the scarifying lancet, and on the other the administration of suitable remedies, thus leaving us almost powerless for relief to our patient.

Besides which we must bear in mind the impossibility of prognosticating the result in any case of convulsions; for once these spasms have begun, who can tell where they will stop? In many cases the nerves once put to their utmost tension seem to keep up their irritability, and are ready to start the muscular contractions from the slightest source of irritation either external or internal. In such cases the usual remedies or appliances are really of very little service, and after repeated attacks the patient dies exhausted, unless speedily relieved.

But what will be said of those cases in which little or no interval to the spasms ever takes place, and the patient dies utterly exhausted without the slightest remission in the distressing symptoms?

In such cases the attempt to administer remedies by the mouth is utterly futile, and from observation of a number of such cases it has appeared to me that any movement of the patient has acted as an additional source of irritation, and that counter-irritation, and even leeching, have produced the same effect. And indeed when we take into account the intensely susceptible state of the nervous system which, in such cases, may be compared to a cord stretched to its utmost capacity and ready to snap by the slightest touch, we cannot wonder that any irritation, even the very slightest, is injurious.

In such instances, almost the only avenue of communication, so to speak, with the interior of the system is through the action of respiration.

If, then, we have a remedy which is powerful sedative of the excito-motary nerves, and which can be safely introduced into the system by means of inhalation, and if such a remedy is had recourse to, before the body is completely exhausted, we at once control the nervous twitching or more severe convulsions and thus give time for throwing off the source of irritation, or we ally the induced over-excitability of the nervous system, and give time for the calming down of the tumult produced.

Such a remedy, I believe, exists ready to our hands in the shape of chloroform, administered by inhalation.

But, without any further preface, let me relate five cases in support of this view: J. R., a boy aged seven months, with a large and very irregular head, child of a very scrofulous mother but a healthy father, had several slight attacks, marked by fixing of the eyes producing the appearance of a continued stare, was seen by me in July, 1859, for convulsions in the form of twitchings, confined at first to the muscles of the face but latterly to both arms. When I first saw the patient he was slightly flushed in the face and the twitchings about the mouth were frequent. His eyes were fixed. He had cut no teeth, but the gums were hot, swollen and dry. The pulse was small and irritable and almost past being counted—fully two hundred a minute. The bowels were also in an irritable state; motions frequent, green and slimy.

The gums were freely cut and remedies administered to relieve the irritation of the bowels as well as of the system. These were accompanied by synapisms to the abdomen. These remedies were continued for three days, during which time the child had several attacks daily, each attack increasing in duration and intensity, and the spasms frequently extending to one or both arms, and this although the gums were repeatedly lanced and although the intestinal irritation had wholly disappeared. On the fourth day the anxiety of the parents was very great, and I was abjured by them to do something for the relief of their boy, (whom by this time they had given up as lost) even if it afforded no prospects of a cure.

Having seen, some months before, a case, reported by Sir James Simpson of Edinburgh to the Obstetrical Society there, in which he had kept a child for nearly a week under the influence of chloroform, allowing it only to awake occasionally for the purpose of having it fed, and which resulted in the complete recovery of the child, I had no hesitation in recommending the administration of chloroform by inhalation in the case of J. R. The parents, anxious to see their child relieved of the spasms, at almost any risk, consented.

After a few inhalations the spasms and twitchings gradually subsided, and in five minutes they had completely disappeared and the child had the appearance of being in a quiet slumber. This was about two o'clock in the afternoon, the child awoke about four, sucked readily, and remained free from spasms until next morning, when it had a recurrence of them, but this time not so severe. The child had a return of the spasms three or four times every twenty-four hours for four days. Each time they were stopped by the inhalation of chloroform, and they gradually diminished in intensity and duration till the last one on the fourth day was merely a slight twitching about the mouth and turning up of the eyes, after which the spasms completely disappeared.

After the second administration of the remedy I instructed the father, who was a very intelligent man, how to give it himself, and he continued to do so during the rest of the time, I seeing the child twice daily. In all, the child took about two ounces of chloroform. This family remained under my care for three month, during which time the child continued well and cut several teeth.

Case No. 2.

E. Allen, a girl. aged four years, apparently healthy, although both father and mother look delicate. She never had an attack of convulsions till the present, which took place during the night of the 23rd July 1861. She had been at a Sabbath school pic-nic on the 22nd, which was a very hot day, was exposed a good deal to the sun, and had partaken freely of half-cooked cakes with dried currants. Her mother was awoke about one in the morning by a peculiar noise and found her child in convulsion; the right side of the face, and the arm and leg of the same side being most affected. In about an hour I saw the patient, she had vomited a little of the cakes and dried fruit, and had had two copious and very fetid alvine evacuations. She had been put into a warm bath. This was repeated with the application of cold to the head, a copious enema, which, however, was not retained any time, administered, synapisms applied to the abdomen, but with no relief to the symptoms. Indeed they rather appeared to increase in intensity. All this time there was powerful closing of the jaws, and no indication of swallowing when liquids where put into the mouth. The pulse was past being counted.

Not having the chloroform with me I despatched the father for it, and in about an hour and a-half from the time she was discovered in the fit began its administration, by inhalation. In ten minutes the spasms had entirely ceased and the child appeared to be in a natural slumber. The remedy was administered twice in the next hour on her appearing to awake. I remained with her two hours longer, and left her in a sound sleep, from which I learned that she awoke about eight o'clock refreshed and ready for her breakfast. During my forenoon visitation I called and found her playing about the house with no trace of the severe ordeal to which her system has been subjected during the night. She had no other remedy, yet she continued well, and I saw her the other day, a stout healthy girl.

Case No. 3.

A. Notz, a large, flabby-looking girl of three and a-half-years, had twice been attended by me during teething for attacks of convulsions, accompanied by gastric derangement. These attacks were slight and she recovered each time in two or three days.

July 27th, 1862—I was called to her about eight in the evening. She had been at a friend's house all day and had eaten a considerable number of unripe plums. She came home complaining only of a little sickness at stomach, was put to bed about seven o'clock and awoke vomiting, and was quickly seized with convulsions. The symptoms were much the same as in the last case, besides which, the evacuations, which were also very offensive, contained what her father called *a hat full* of half digested unripe plums. The jaws were also set and deglutition impossible. Before my arrival she had been put into a hot bath. There was no abatement of the spasms until she was brought fully under the influence of chloroform, which I carried with me in this instance, as I had come to consider it a valuable remedy. After she had been brought fully under its influence the inhalation was stopped, and not resumed again till she was allowed to awake, which she did in half an hour, apparently well. In few minutes, however, the spasms returned, but were immediately checked by the remedy. This was repeated frequently during the next four hours, and at two in the morning I left her in a natural slumber into which she had laspsed from the anaethesia, and from which she awoke about her usual time in the morning, almost well, but suffering from great lassitude and prostration. This continued for several days with slight febrile attacks. These were treated with alteratives and tonics, and in a few days she was well. She has had two attacks of febris infantum remittens since, but no return of the convulsions.

### Case No. 4.

E. T., aged three years and two months, was in the garden playing with other children on Oct. 8th, 1862, till about four P. M., when she came into the house in a stupid like state, and it was thought that she must have fallen from a low branch of a tree on which she had been put by one of her brothers. I saw her in less than five minutes, when she seemed to be getting more sensible, but was still unable to speak. When asked what ailed her she put her hand to her head. She could not be got to swallow, and as she seemed improving I told them to watch her. In about a half an hour, and without her becoming quite conscious or able to speak, she was seized with twitchings of the facial muscles and fixing of the eyes and jaws. There was also clenching of the fists with inversion of the thumbs; she had had a slight alvine evacuation but it was not fetid. Pulse quick.

I at once administered chloroform in the usual way, and kept it up for about two hours. The spasms yielded to the remedy at once, but recurred slightly when it was discontinued long.

At the end of the time mentioned she had lapsed into a natural slumber, which continued all night and from which she awoke apparently well. Next day she went about the house as usual, and was only restrained, by the anxiety of of her parents, from entering into the ordinary excitements of a playful girl of her age. She has had whooping cough since, but no return of the convulsions.

CASE NO. 5.

On 29th April last I delivered Mrs. Jones of her first child, a female, by the aid of instruments, having first administered chloroform. The child was small, but throve well till June 13th, when, without any apparent cause, it was seized with fixing of the eyes and twitchings of the face. I saw it soon after, and while there it had a fierce convulsive attack. The child being only seven weeks old, and I never having heard of the administration of chloroform to so young a subject, at first hesitated, but as the convulsions were very severe and recurred so rapidly as to prevent the administration of medicines or nutriment, I was forced to the alternative, and administered chlorform inhalations very cautiously, and was delighted to find that, although it did not control the convulsions so rapidly nor for such a length of time as in the other cases, still it gave sufficient space between the paroxysms to admit of the administration of food and medicines, and for two days the child continued almost free from convulsions. On the following night, however, I was summoned again, the convulsions having set in with renewed severity. Chloroform was again administered with good effect; but it was now evident that head symptoms had supervened, and I cast about me for another remedy with which to combat these, the chloroform being still continued at intervals to check the spasms. It must be confessed that I was somewhat at sea as to the proper dose of the bromide of potassium for so tender an age as seven weeks, and thought it best to begin cautiously, with three grains in simple solution. The effect being beneficial the dose was gradually increased every two hours, till six and ultimately ten grains were reached. The convulsions ceased in the two days, but the child lapsed gradually into a semi-comotose condition, from which it recovered in a few days to reveal the mortifying fact that total blindness had supervened. Counter irritation to the scalp, by means of croton oil, as recommended in a recent number of Edinburgh *Medical and Surgical Journal*, had the most happy effect in removing the blindness, and the child was soon restored to the use of all its faculties, the only disagreeable symptoms remaining being a double strabismus. A few days ago I saw the child, and it looked intelligent, plump and healthy.

REMARKS.

In remarking on these cases I may say that they represent very well the class of cases of greatest severity which occur to medical men in their practice among children, and I have little doubt that but for the administration of the chloroform one or more of them would have proved fatal. This I say from my recollection of many cases seen during a connection of over a quarter of a century with the Medical Profession. Taking a restrospective glancein to that period the mind reverts with painful interest to several cases of convulsions in children, suddenly supervening after some error of diet, either in quantity, or during the period of teething, without any organic completion, and resembling in their mode of attacks, one or other ot the five successful cases given above, so closely as to bring conviction to my mind that had we known the mode of treatment specified, a cure would have been the result in all or nearly all.

MODUS OPERANDI.

As to the *modus operandi* I have already stated my belief that, in cases such as are given above—and I believe that they are the only class of cases in which we may look for success from this mode of treatment—the remedy acts by keeping the nervous system, and more especially the spinal portion, in a quiescent or even torpid state till such time as the irritation shall have passed away, and the system is left in something like its nomal condition to resume its functions.

M. Billard has shown that affections of the spinal cord are more frequent in young children than those of the brain; and M. Olivier states that the symptoms of the former are much better marked than those of the latter, and I look upon these convulsions as being entirely connected with derangement of the functions of the spinal marrow, and being the manifestation of reflex action produced by indirect irritation of the excito-motary system, and this view is, I believe, upheld by the action of chloroform in the cases above noted, as, had they been primarily connected with derangement of the sensorium, they would have shown different results.

UNSUCCESSFUL CASES.

It will naturally be asked is there no dark side to this picture, no record of unsuccessful cases to be related which might shake one's faith in the potency of this remedy in convulsions. In answer I say that, in all kindred cases in which I administered chloroform, it has been invariably successful, and in only one case was it given unsuccessfully, and that, as was anticipated, proved fatal. During the time over which those cases spread I have seen frequent cases of convulsions in children, but they were either so slight and transient that they required little or no treatment, or they depend on organic disease of the brain and were not adapted for the remedy, so that it was not considered proper to try it except in the one case, which I will now briefly relate.

Case 6.

E. M., a delicate girl of eleven years, was also at the Sabbath School pic-nic which we have mentioned in case No. 2. The day was sultry and the heat very powerful. Unlike the child Allen, she had fastened all day with exception of large drafts of water. Indeed, she was probably ill before going to the pic-nic, and on coming home took to her bed, complaining of headache, accompanied with chills, followed by fever. An officious neighbor pronounced it ague, and the poor child was dosed with quinine for a week, during which time the symptoms continued to increase in urgency, owing, accordingly to the aforesaid neighbor, to the doses of the bitter being too small. These were increased with no better results, and about the end of the week when I was called in, she was in the last stages of Hydrocephalus, como and convulsions being present. The convulsions were so distressing to the parents that they begged of me to give her something to allay them. I told them that there was no hope of her recovery, and that the only remedy I knew of as likely to stop the spasms was chloroform, inhaled. They consented to its administration, and, merely with a view of relieving suffering and not with any hope of cure, it was given. The spasms where held in check while the patient was under the influence of the drug, but recurred with equal violence when it ceased to act. For two days she lingered in this way, gradually getting weaker, till death supervened.

I believe in this case the chloroform did no harm, and it certainly seemed to relieve the patient's sufferings; at all events, the feelings of the parents and friends were spared the witnessing of the distressing spasms. Of course this case cannot be brought forward against the use of chloroform in cases such as this paper refers to.

Since tbe commencement of this treatment I have notes of other twenty-two similar cases, treated in the same way, and invariably with success. I may mention that I always give the chloroform by means of a thin handkerchief or towel folded up so as to form a hollow cone in the hand.

I have since used it *guttatim*, after the method introduced by Dr. Moir of Edingurgh, as related by Sir James Simpson in the Edingurgh *Medical Journal* for 1863, and since simplified and rendered more certain by Dr. A. M. Roseburgh, viz.: that of covering the face with a single fold of a towel and dropping the anæsthetic on it very slowly.

In conclusion I would strongly recommend the treatment of convulsions of this class by chloroform inhalation, and will be glad to get the views of my medical brethren on the subject.—*Canada Medical Journal.*

